# Supplementary material for: Differential Hebbian learning with time-continuous signals for active noise reduction
Source: PLoS One. 2022 May 26;17(5):e0266679. doi: 10.1371/journal.pone.0266679 (PMC9135254; doi:10.1371/journal.pone.0266679)
Supplement: Attachment — Submitted filename: LoC02.pdf [file pone.0266679.s001.pdf]

PONE-D-22-08630 List of Changes

Thanks for all comments. We have addressed them all and provide here the details. All changes are marked in the text in red color.

Reviewer #1:

The main contribution of this paper is the show that it is possible to use a learning rule derived from spike timing dependent plasticity found in biological synapses for active noise active noise reduction. The proposed Differential Hebbian learning rule successfully reduces the noise under a range of different simulated configurations. In addition to pure noise cancellation, the method also allows for specific parts of an incoming noisy signal to pass through while filtering out the noise.

The paper show cases the practical potential of a bio-inspired learning rule that quickly adapts to changing noise reduction needs. The variety of different configurations used to test the methods highly strengthens its credibility for potential applications.
The authors provide thorough implementation details to aid replicabillity of the study.

Minor Suggestions:

- In the Results & Discussion section, a specifics of the series of experiments are introduced. The paper might have been a bit easier to read had the different configurations been listed in a section called Experiments (or similar). Then the Results & Discussion section could be reserved to presenting and discussing the results of the experiments.

In the Methods section, we have now added a table where we describe and number the different experiments, their goals and their basic setup referring to the figures to come. In the results section, the experiments are then numbered accordingly.

- In the Conclusion, the authors refer to a series of older papers (line 228, line 428). Perhaps it would make sense to include these references in the introduction instead, to give the reader more context early on.

These papers are not of immediate relevance to the introductory motivation of this study. Hence, we think it is better to keep them in the Conclusions section not to disrupt the train of thought in the Introduction by this. (No text change).

- In line 22, I think “had” should be replaced with “have”.

Thanks, done!

- Sentence starting in line 29 could benefit from a reformulation, perhaps with a deletion of “In general,”.

Thanks, we reformulated this.

- The sentence starting in line 231: “Thus, the fact that the later signal x0 is used as the error for learning leads in those older studies, but also in the here-introduced system, to convergence.” is a bit hard to make sense of with the interposed sentences, and could perhaps be reformulated.

We have reformulated this, too.

- Perhaps I don't know the terminology around active noise reduction well enough, but it is not entirely clear to me what is meant by "at a high level" in sentences in line 135 and line 156.

We changed this statement to make this clearer.

Reviewer #2:

The abbreviations ICO and ISO are not defined.

We have now defined them where they had been mentioned first.

A section on intuition regarding what ICO is doing and how it counteracts the noise would be useful.

We have added a paragraph in the Methods section that gives some intuition on how ICO works.

A little more detail in the learning rule section and noise reduction mechanism section would help the reader follow the arguments more easily.

See also response above. We have added more information about the learning mechanisms. And we have now also added a bit more information about the noise reduction mechanism in the Methods section.
